# Honoring Lihong V. Wang, a pioneer in biomedical optics

**DOI:** 10.1117/1.JBO.29.S1.S11501

**Published:** 2023-12-12

**Authors:** Steven L. Jacques

**Affiliations:** University of Washington, Department of Bioengineering, Seattle, Washington, United States

**Keywords:** optical, spectroscopy, photoacoustic

## Abstract

A pioneer in optics based on his development of novel optical imaging techniques and acknowledged by a long list of honors, Lihong V. Wang is a model for the aspiring young student or investigator pursuing a career in the rapidly expanding field of biomedical optics and biophotonics.

## 1 Introduction

**Fig. 1:**
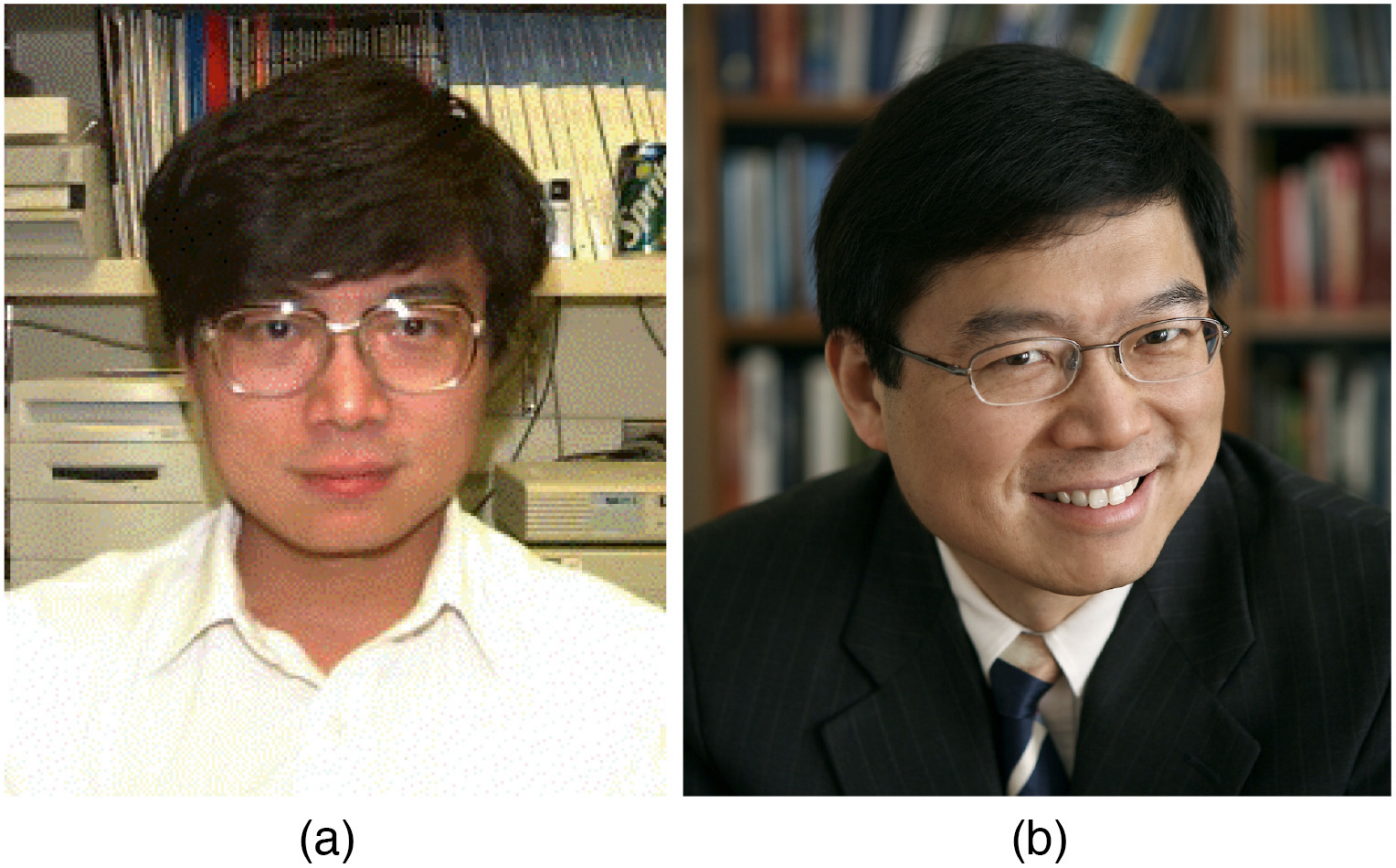
Lihong V. Wang (a) as an assistant professor,^2^ with permission from Ref. 5 and (b) now at Caltech,^3^ with permission from COIL, CalTech.

When asked during an interview,^1^ “What is the best descriptor for his research career?,” Lihong Wang answered “Discovery.” Indeed, his career has coincided with the early development of optical methods for imaging biological tissues to characterize normal versus pathologic tissue, monitor delivery and effects of drug administration, and design both devices and clinical protocols for photo-therapeutic treatments. The creation of new optical tools enabled the ability to view tissues in new ways, catalyzing the discovery of biological behavior by the photomedicine community. When asked “What is your advice to students entering this field?,” he answered “Passion,” advising students to have great passion for their research project. I worked with Lihong during his early career as a postdoc and assistant professor (Fig. 1) and personally experienced this excitement of discovery and passion.

Lihong received his BS and MS degrees in optics from Huazhong University of Science and Technology, in the National Key Laboratory of Laser Technology, Wuhan, China. He traveled to Houston, Texas, to receive his PhD in electrical engineering from Rice University in 1991. He was working with a team of advisors, Frank K. Tittel (former department chair), Robert F. Curl, and Richard E. Smalley (both Nobel laureates for their work on the buckminsterfullerene molecule). His PhD work was on gallium arsenide clusters. In the *Laser Focus World* interview,^1^ Lihong described how he worked closely with this team, meeting throughout each week on the project. This early experience with teamwork was to have a strong influence on his career. Frank told me that he first met Lihong fresh from China, standing on the curb with his suitcase waiting to be picked up. Frank marveled at how that young Lihong developed into a productive investigator and leader in our field.

He then moved to the University of Texas M.D. Anderson Cancer Center (UTMDACC), Laser Biology Research Laboratory as a postdoc from 1991 to 1993 and assistant professor from 1993 to 1996. There he participated in another team that worked closely together: Andreas Hielscher (Rice graduate student, currently Biomedical Engineering Department Chair at New York University), Sharon Thompson M.D. (pathologist), Alexander Oraevsky [postdoc, with career in photoacoustic imaging (PAI)], and Steven Jacques (lab director) in the early development of light transport and laser–tissue interactions in medicine and biology. He repackaged the lab’s Monte Carlo simulations of photon transport (Marleen Keijzer, Scott Prahl, Steve Jacques) into the ANSI-standard C-code MCML.c, in which he reorganized the program as an executable that read an input file to specify the particulars of a simulation run, enabled multiple layers of media with different optical properties and refractive indices, and improved the subroutine for handling Fresnel reflections/transmissions at boundaries (cited more than 4369 times as of September 2023 according to Google Scholar; an open-source simulation package, it has become a standard in the field of biomedical optics).^4^ Lihong set up the lab website and posted the source code for MCML.c. At that time, the internet was just beginning to emerge in the public domain. Websites were not yet common, and his effort was an innovative new step. The website grew and boosted the international profile of the laboratory. Scott Prahl later reorganized the website into the Oregon Medical Laser Center website^5^ that is widely used.

Lihong supervised an undergraduate, Dawn Stephens, on a study of polarized light reflectance from a scattering medium illuminated with a point source of linearly polarized light. They studied the movement of the “Maltese Cross,” the cross pattern of reflectance from milk irradiated with a linearly polarized HeNe laser while observed through a second linear polarizer as it was rotated. The cross moved only half as fast as the polarizer rotated. It was intriguing. Jacques asked that the experiment be done on skin, which showed a very small, not quite visible Maltese Cross. This result indicated that polarized light imaging (PLI), which images the difference between co-polarized and cross-polarized images (i.e., the Q of the IQUV Stokes vector), would not suffer significant blurring. Hence, this undergraduate study was the inspiration for PLI, which Jacques continued to develop for imaging superficial tissue layers. Lihong would later publish an optical coherence tomography (OCT) based measurement of the Mueller matrix of tissues (discussed later).

The laboratory also did early work on photoacoustics using pulsed ns lasers. Alexander Oraevsky had trained in Russia in photoacoustics and came to the lab as a postdoc. Alexander worked on piezoelectric detection of pressure waves generated by pulsed lasers in absorbing media, converting the signals into the optical properties of absorption and scattering. This early work was exciting for all. Lihong was promoted to assistant professor at UTMDACC. Lihong conducted an early test of ultrasound-modulated optical tomography, in which an ultrasound wave generator was focused to a focal point within a light-scattering medium. Broad beam light was delivered to the medium and transmitted light was detected. The transmitted light signal that was modulated at the ultrasound frequency (∼1  MHz) indicated the absorption of light at the ultrasound focal point. As the local point was translated within the medium, the spatial distribution of absorption was specified, yielding a 3D tomographic image. Lihong would later work on RF-acoustic imaging (see below).

In 1996, Lihong was recruited to Texas A&M University (TAMU), as an assistant professor in biomedical engineering, eventually rising to the Royce E. Wisenbaker II Endowed Professor of Engineering. One of his early studies was the OCT-based Mueller matrix which converted an input Stokes vector description of incident polarized light into the output Stokes vector of transmitted polarized light, hence characterizing the tissue (or medium) by its ability to alter polarized light. At the time, OCT was the hottest topic in our field, and this study was quite innovative.^6^ Lihong also conducted RF-based acoustic imaging, using a pulse RF field to impart energy deposition in the water of a sample. The resulting thermal expansion launched an acoustic wave, and time-resolved detection of the acoustic waves allowed back-projection to map the distribution of water in the sample. He also pursued PAI at TAMU.

A big step in his career was when Lihong published an *in vivo* photoacoustic image of the blood vessels of a mouse through the intact skull and scalp, which garnered much attention.^7^ The resolution of the blood vessels was impressive, and stimulating the whiskers modulated the blood flow to demonstrate functional imaging. Lihong improved the method by the design of a confocal photoacoustic imager.^8^ A pulsed ns laser was bounced off a circular ring mirror to focus light to a focal point within the tissue. The resulting acoustic wave was collected through an acoustic lens in the center of the circular mirror, a plastic material shaped to convert the expanding spherical acoustic wave from the focal point into a broad flat beam, i.e., a Fourier transform of the point source. This flat beam struck a piezo-electric film that detected the signal. Hence, light was delivered to a focal point and the piezo-film detected from the focal point, i.e., confocal detection. The sample could then be scanned relative to the focal point to yield a spatial map of light absorption within the sample. This was an innovative technique and a clever design. Lihong and Alexander Oraevsky established the “Photons plus Ultrasound: Imaging and Sensing” conference at the SPIE Photonics West BiOS symposium, which quickly grew into one of the largest conferences in BiOS.

**Fig. 2:**
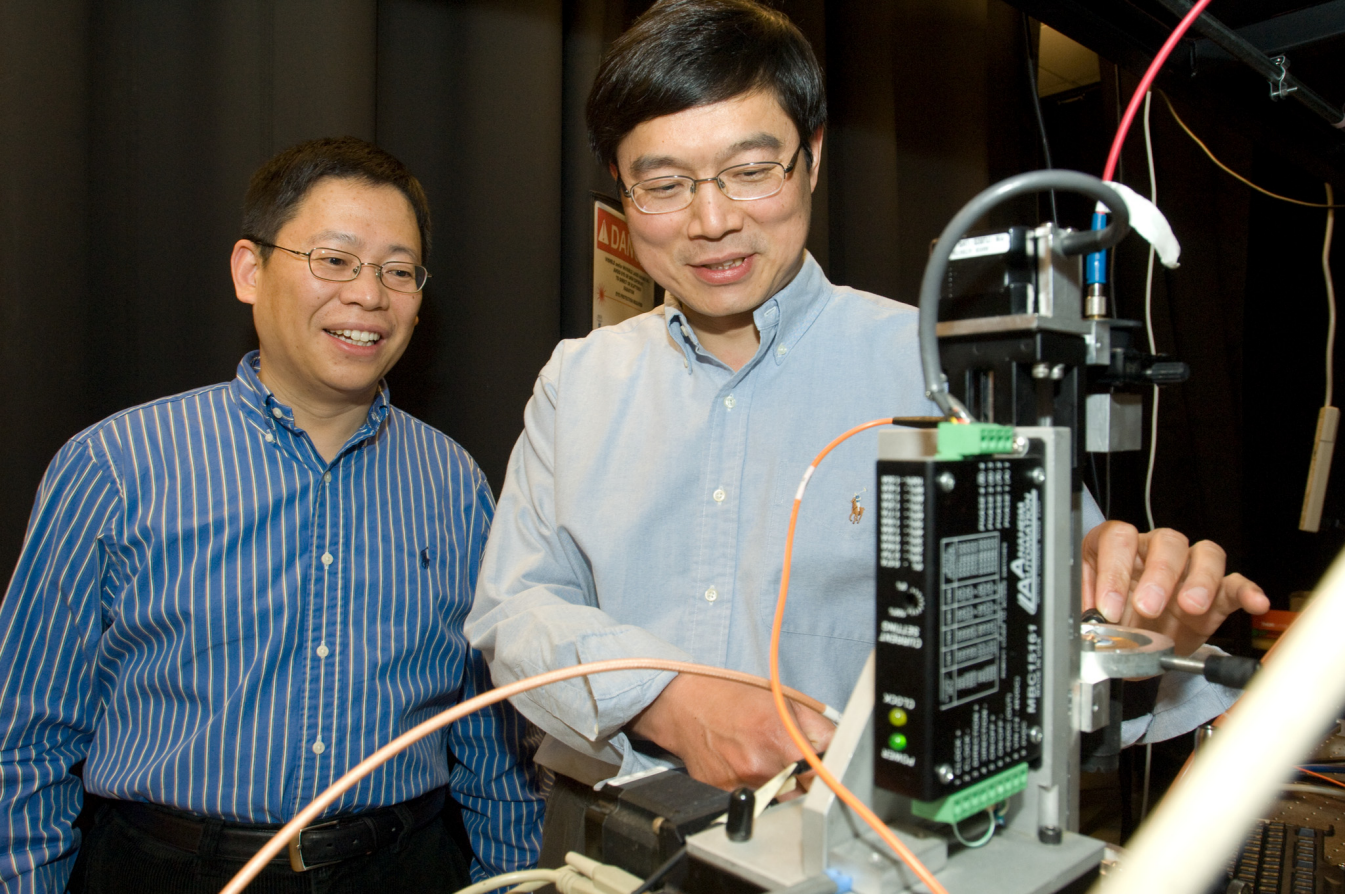
Younan Xia and Lihong Wang work on PAI of sentinel lymph nodes to guide lymph node biopsy procedures for breast cancer patients. (Reproduced with permission from WUSTL.)

In 2006, Lihong was recruited to Washington University in St. Louis (WUSTL) as the Gene K. Beare Distinguished Professor of Biomedical Engineering. His work on PAI refined the technique, using high repetition rate ns lasers to increase the speed and quality of images. One project was the using of PAI to detect sentinel lymph nodes in the axial nodes (arm pits) of patients with breast cancer (Fig. 2). The concept was to inject an absorbing dye, e.g., indocyanine green or methylene blue, into the cancerous tumor and watch for the arrival of dye in the axial nodes via blood vessels draining the breast. Then, surgical excision could remove those sentinel lymph nodes and avoid removal of the many unaffected lymph nodes, which greatly improves the quality-of-life outcome for patients. Work continues in various centers (e.g., the Netherlands, China, and the United States) on this method. Another project was photoacoustic microscopy (PAM). Images of melanoma showed a pigmented melanoma and the surrounding blood vessels.^9^ Dual-wavelength PAM could also image the oxygenation of individual blood vessels.^10^ Implementing PAM with a pulsed UV laser used DNA absorption to image the spatial distribution of nuclei in cells.^11^

Lihong also contributed to time-reversed ultrasonically encoded optical focusing, in which an ultrasound modulated focus served as a “star” for characterizing the time-resolved escape of photons from a point source within a medium. This characterization of photon escape could then guide the wavefront of a beam delivered to the surface in order to focus photons to a focal point, thereby correcting for light scattering. This method shared similarities to his earlier work on light transmission through an ultrasound modulated focus.

This work on PAI as a multi-scale imaging modality from the micro-scale to the macro-scale was summarized by Yao, Xia, and Wang in their review paper.^12^ While optical fluorescence and reflectance confocal imaging, second-harmonic generation, and OCT can image superficially, and ultrasound, x-ray, and NMR tomography could image macroscopically, there was a middle range covered by PAI. However, PAI can also do both microscopic and macroscopic imaging, providing a modality for noninvasive multiscale biochemical, functional, and molecular imaging from organelles to humans at high resolution. By promulgating the multi-scale opportunity for PAI, Lihong has motivated a large community of investigators to continue development of this important imaging modality. In their review (see Fig. 1 in Ref. 12), the multiscale was summarized:

1. Cellular imaging [up to 100  μm, the aberration limit (=1/scattering coefficient)]. Includes conventional planar optical microscopy.
2. Superficial imaging (e.g., skin) [100  μm to 1 mm, the diffusion limit (−10/scattering coefficient)]. Includes confocal and two-photon microscopy, OCT, and optical-resolution photoacoustic tomography (PAT).
3. Organ imaging [1 mm to 10 cm, the dissipation limit (=10/attenuation coefficient)]. Includes diffuse optical tomography, ultrasound-modulated optical tomography, and acoustic-resolution PAT.
4. Human imaging [10 cm to 1 m, the absorption limit (=10/absorption coefficient)]. Includes wavefront engineering with internal guidestars.

**Fig. 3:**
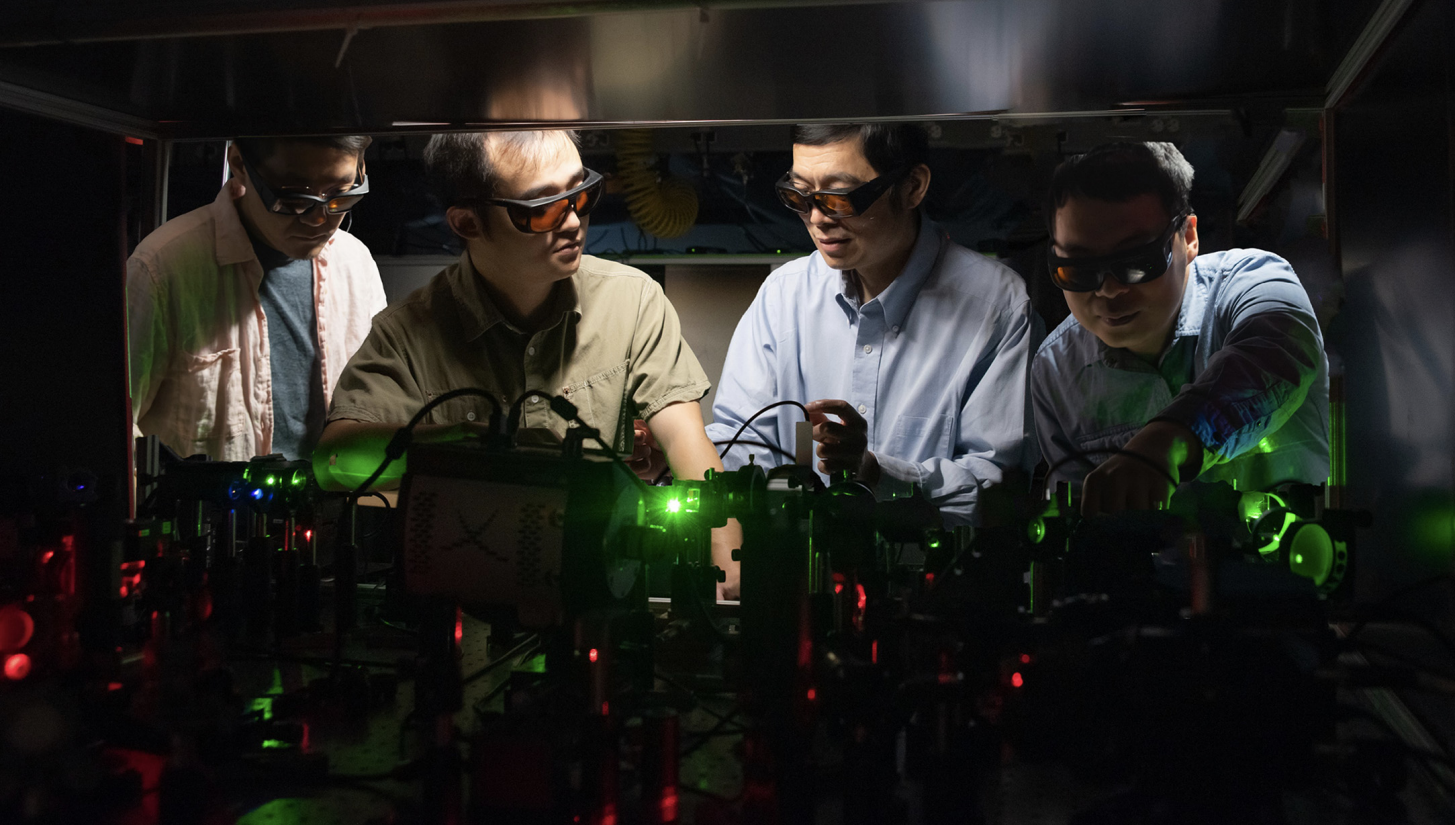
Working on quantum optics in the laser lab. Lihong is second from right. (Reproduced with permission from COIL, CalTech.)

Continuing to work on time-resolved photon transport, Lihong’s WUSTL lab developed light-speed compressed ultrafast photography, which created a video sequence of a laser pulse propagating based on a single laser pulse.^13^ Using a streak camera and compressed sensing techniques, this “fast camera” showed a laser pulse being refracted by a medium. Such single-shot video sequences were mind-blowing, and this project made big headlines. This innovation received a lot of press.

In 2017, Lihong was recruited to the California Institute of Technology (Caltech) as the Bren Professor of Medical Engineering and Electrical Engineering and now is a chair of the Andrew and Peggy Cherng Department of Medical Engineering. His Caltech Optical Imaging Laboratory (COIL) is one of the largest at Caltech. The current work at COIL includes computed ultrafast photography, PAT, ultrasound-modulated (acousto-) optical tomography (UOT), wavefront shaping (WFS), and quantum optics, including imaging (Fig. 3). The COIL website^14^ provides an up-to-date summary of projects and recent publications.

Lihong has to-date published 592 peer-reviewed papers and 69 patents and disclosures. He has supervised 106 research faculty, research associates, postdocs, and staff, and 60 doctoral students. He has so far given 595 invited talks around the world. He served as editor-in-chief of the *Journal of Biomedical Optics* from 2010 to 2017. Lihong Wang has received many honors, and a partial list is presented below. Lihong has been actively engaged in the biomedical optics community, both in education and service. The key point here for a student or young investigator striving to succeed is “engage with one’s community.”

December 2020: Life Fellow, National Academy of Inventors (NAI).
October 2019: Life Fellow, American Association for the Advancement of Science (AAAS).
May 2019: Life Fellow, International Academy of Medical and Biological Engineering (IAMBE).
February 2018: Life Member, National Academy of Engineering. “For inventions in photoacoustic microscopy enabling functional, metabolic, and molecular imaging *in vivo*.”
February 2018: Michael S. Feld Biophotonics Award, OSA (now Optica). “For inventing the world’s fastest two-dimensional receive-only camera and enabling real-time imaging of the fastest phenomena such as light propagation and fluorescence decay.”
September 2015: Senior Prize of the International Photoacoustic and Photothermal Association (IPPA). “For seminal technical contributions and visionary scientific leadership in the field of biomedical photoacoustic imaging.”
April 2015: Britton Chance Biomedical Optics Award, SPIE. “For pioneering technical contributions and visionary leadership in the development and application of photoacoustic tomography, photoacoustic microscopy, and photon transport modeling.”
August 2014: Fellow, Electromagnetics Academy.
August 2014: IEEE Biomedical Engineering Award. “For pioneering photoacoustic tomography.”
December 2013: Honorary Doctorate, Faculty of Engineering, Lund University, Lund, Sweden. “For groundbreaking contributions to photoacoustic tomography.”
May 2011: Technical Achievement Award, IEEE EMBS. “For seminal contributions to ultrasonically enabled biophotonic imaging and modeling of photon transport in biological tissue.”
March 2011: Induction into the GRC Hall of Fame, Gordon Research Conferences.
February 2011: C. E. K. Mees Medal, OSA. “For seminal contributions to photoacoustic tomography and Monte Carlo modeling of photon transport in biological tissues and for leadership in the international biophotonics community.”
March 2010: Joseph W. Goodman Book Writing Award, OSA & SPIE. *Biomedical Optics: Principles and Imaging* by Lihong V. Wang and Hsin-i Wu, Wiley, 2007.

Perhaps the most important emphasis of this article should be how well Lihong has developed teams that are focused on discovery. One recognizes a continuous line of passion for discovery, from his early work at Rice University through to the present COIL program at CalTech. He has inspired his students and trainees with his passion and shared with them the joy of discovery. The impact of his trainees will greatly expand the accomplishments of his own career. Lihong Wang serves as an excellent model for a young investigator.

**Fig. 4:**
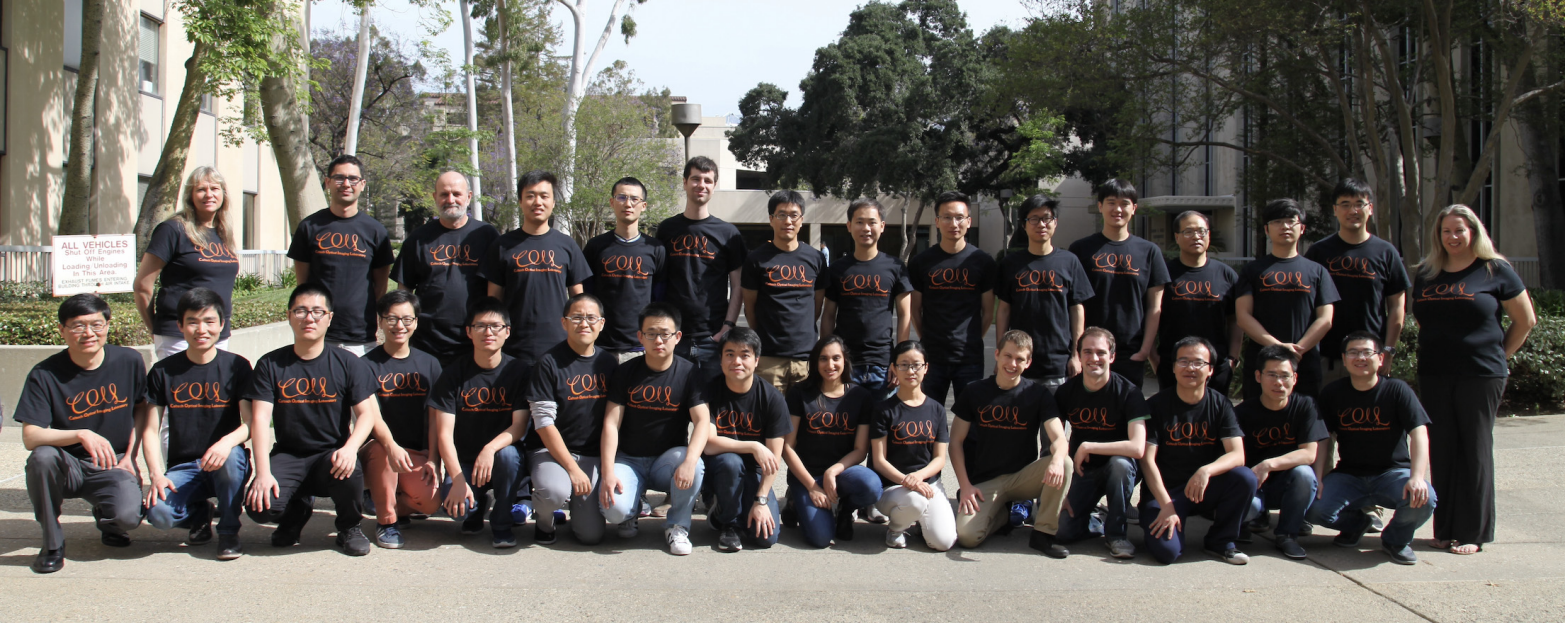
The COIL. Lihong Wang at lower left. (Reproduced with permission from COIL, CalTech.)
